# Clinical characteristics of coronavirus disease (COVID-19) early findings from a teaching hospital in Pavia, North Italy, 21 to 28 February 2020

**DOI:** 10.2807/1560-7917.ES.2020.25.16.2000460

**Published:** 2020-04-23

**Authors:** Marta Colaneri, Paolo Sacchi, Valentina Zuccaro, Simona Biscarini, Michele Sachs, Silvia Roda, Teresa Chiara Pieri, Pietro Valsecchi, Antonio Piralla, Elena Seminari, Angela Di Matteo, Stefano Novati, Laura Maiocchi, Layla Pagnucco, Marcello Tirani, Fausto Baldanti, Francesco Mojoli, Stefano Perlini, Raffaele Bruno, the COVID19 IRCCS San Matteo Pavia Task Force

**Affiliations:** 1 Division of Infectious Diseases I, Fondazione IRCCS Policlinico San Matteo, Pavia, Italy; 2 Department of Hygiene and Preventive Medicine, Health Protection Agency of Pavia, Pavia, Italy; 3 Directorate General for Health, Lombardy Region, Milano, Italy; 4 Molecular Virology Unit, Microbiology and Virology Department, Pavia, Italy; 5 Department of Clinical, Surgical, Diagnostic, and Pediatric Sciences, University of Pavia, Pavia, Italy; 6 Anesthesia and Intensive Care, Emergency Department, Fondazione IRCCS Policlinico S. Matteo, Pavia, Italy; 7 Anesthesia, Intensive Care and Pain Therapy, Fondazione IRCCS Policlinico San Matteo, Pavia, Italy; 8 Emergency Department, Fondazione IRCCS Policlinico San Matteo, Pavia, Italy; 9 Department of Internal Medicine and Therapeutics, University of Pavia, Pavia, Italy; 10 The members of the COVID19 IRCCS San Matteo Pavia Task Force are listed at the end of the article

**Keywords:** retrospective study, COVID-19, SARS-CoV-2, severe disease, LDH

## Abstract

We describe clinical characteristics, treatments and outcomes of 44 Caucasian patients with coronavirus disease (COVID-19) at a single hospital in Pavia, Italy, from 21–28 February 2020, at the beginning of the outbreak in Europe. Seventeen patients developed severe disease, two died. After a median of 6 days, 14 patients were discharged from hospital. Predictors of lower odds of discharge were age > 65 years, antiviral treatment and for severe disease, lactate dehydrogenase > 300 mg/dL.

Severe acute respiratory syndrome coronavirus-2 (SARS-CoV-2) is an emerging virus recently detected and associated with a severe respiratory disease, named coronavirus disease (COVID-19), which has been declared a pandemic disease by the World Health Organization (WHO) on 11 March 2020 [1].

As at 20 April 2020, 1,149,071 confirmed cases of COVID 19 were reported in Europe, of which 181,228 were in Italy [2].

To date, while there are numerous studies describing clinical characteristics of COVID-19 patients from China [3-6], there are only few published from European countries. Here we report early findings on clinical presentation, treatment and clinical outcomes of patients with COVID-19 in a large teaching hospital in Pavia, Lombardy region, northern Italy, as well as preliminary analyses of predictors of discharge status and of developing severe disease.

## Data extraction and analysis

We extracted data from medical records of all consecutive patients admitted with a diagnosis of COVID-19, between 21 and 28 February 2020 and followed-up until 4 March 2020. We collected demographic data (sex and age), clinical data (symptoms on admission, comorbidities and chest X-ray results), laboratory tests and treatment data (use of antivirals, antibiotic drugs and oxygen support). Diagnosis of COVID-19 was confirmed by positive real-time reverse transcriptase PCR from nasal swabs, which were analysed by the molecular virology unit of our hospital according to the WHO guidelines and protocol by Corman et al. [7].

Severe disease was defined as requirement for high-flow oxygen support. Low (cannula and simple masks) and high-flow (Venturi and reservoir masks) oxygen support were provided when hypoxia defined as peripheral oxygen saturation < 90% was detected.

Univariate logistic analysis was employed to identify variables associated with hospital discharge and development of severe disease. The multivariable model was built to determine the association between the variables which threshold values were <0.10 at univariate analysis. In this final model, variables with a p value < 0.05 were considered statistically significant.

## Clinical characteristics

From 21 February to 28 February 2020, 44 confirmed cases of COVID-19 were observed. The majority were males (28 males, 16 females). Median age was 67.5 years (range: 10–94 years, interquartile range (IQR): 29.1) and 19 patients were under 65 years old. Clinical signs on admission were fever (n = 40), cough (n = 15 ), dyspnoea (n = 10), diarrhoea (n = 3), weakness (n = 2); confusion, respiratory failure, constipation, chest pain and muscle pain were each reported by one patient (Table 1).

**Table 1:** Clinical characteristics, imaging results and laboratory findings of patients with laboratory-confirmed COVID-19 by disease severity and discharge status, Pavia, northern Italy, 21–28 February 2020 (n = 44)^a^

	Total sample	Stratified by disease severity^b^		Stratified by discharge status^b^	
		Mild disease (n = 27)	Severe disease (n = 17)	Not discharged (n = 30)	Discharged (n = 14)
	n	n	n	n	n
Sex (F)	16	12	4	9	7
Age > 65 years	25	12	13	22	3
Clinical manifestation					
Respiratory frequency > 22/min	16	7	9	14	2
Fever (Body Temperature > 37.5°C)	40	23	17	30	10
Cough	15	10	5	13	2
Dyspnoea	10	6	4	8	2
Diarrhoea	3	1	2	2	1
Weakness	2	2	0	5	1
Chest X-ray					
Interstitial pneumonia	31	17	14	26	5
Comorbidities					
Presence of comorbidities	28	15	13	24	4
Cancer	6	2	4	5	1
Heart disease	11	5	6	8	3
Hypertension	15	10	5	14	1
Diabetes mellitus	7	6	1	6	1
Lung disease	2	2	0	1	1
Chronic hepatitis C	2	1	1	1	1
Laboratory data					
PF ratio < 260 (n = 25)	10	2	8	10	0
CO2 < 35 (n = 25)	16	10	6	16	0
pH < 7.45 (n = 25)	7	5	2	7	0
Leukopenia (white blood cell count < 5,000 cells/mm^3^)	22	13	9	17	5
Lymphopenia (lymphocyte count <1,500 cells/mm^3^)	39	22	17	29	10
Thrombocytopenia (platelets <150,000/ mm^3^)	19	9	10	15	4
CD4^+^ T-cell count < 250 cells/μL	14	8	6	10	4
LDH > 300 mU/mL	15	5	10	13	2
Creatinine > 1.5 mg/dL	2	0	2	2	0
CRP elevation (CRP > 10 mg/dL)	8	3	5	8	0
25 ng/mL	8	3	5	8	0
Therapy					
Antiviral therapy^c^	31	14	17	27	4
Antibiotic therapy^d^	32	17	15	27	5

CO: carbon dioxide; CRP: C-reactive protein; F: female; LDH: lactate dehydrogenase; PCTI: procalcitonin; PF ratio: arterial partial pressure of oxygen/fractional inspired oxygen.

^a^ Follow up as at 4 March 2020.

^b^ Patients were included in the mild disease group if they did not need high-flow oxygen support and in the severe disease group if they were provided with high-flow oxygen support.

^c^ Lopinavir/ritonavir (LPV/r) 200/50 mg twice a day plus hydroxychloroquine 200 mg twice a day.

^d^ Piperacillin/tazobactam and doxycycline.

Remarkably, 16 patients had no underlying comorbidities. The most common comorbidities were hypertension (n = 15), heart disease (n = 11), diabetes mellitus (n = 7), history of cancer (n = 6; five solid carcinomas, one B-cell lymphoma), lung diseases (n = 2) and chronic hepatitis C (n = 2). Only one patient had chronic kidney disease. We observed no cases with known immunodeficiency, either congenital or acquired. Chest X-ray revealed the presence of 31 cases of interstitial pneumonia (n = 31). Table 1 shows laboratory findings on admission. Lymphocytopenia was present in 39 patients, thrombocytopenia was observed in 19, and leukopenia in 22. CD4+ T-cell count was < 250 cells/μL in 14 patients. LDH levels were elevated in 15.

Twenty-five patients with hypoxia underwent an arterial-blood gas test. Assuming a normal arterial oxygen partial pressure to fractional inspired oxygen (PaO2/FiO2 or P/F) ratio in the remaining 19 patients, a P/F ratio less than 260 was found in 10 patients.

Thirteen patients did not receive any antiviral drug, while 31 patients received antiviral treatment (Table 1). Antiviral treatment consisted in lopinavir/ritonavir (LPV/r) 200/50 mg twice a day plus hydroxychloroquine (HCQ) 200 mg twice a day. Antibiotic therapy was started in 32 patients and consisted of piperacillin/tazobactam and doxycycline. Supportive therapy was delivered based on clinical indications.

## Factors associated with clinical outcome

In the univariate analysis for discharge, age > 65 years, interstitial pneumonia, presence of comorbidities, hypertension and antibiotic therapy were significantly associated with lower odds of discharge. In the univariate analysis for disease severity, male sex, respiratory frequency > 22, cancer as comorbidity, thrombocytopenia and arterial partial pressure of oxygen/fractional inspired oxygen (PF) ratio < 260 were significantly associated with higher odds for severe disease.

LDH levels > 300 mU/mL and antiviral therapy were associated with both outcomes (Supplementary material, Table S1).

As at 4 March 2020, 14 patients were discharged from the hospital. In these patients, median time from symptom onset was 12 days (range: 6–30 days, IQR: 6) and median time of hospitalisation was 6 days (range: 4–7 days, IQR: 2). Age over 65 years and antiviral treatment were significantly associated with lower odds of discharge, by multivariable analysis (Table 2). Seventeen patients had severe disease and there were two fatalities. LDH levels > 300 mU/mL were significantly associated with higher odds of severe disease, by multivariable analysis (Table 2).

**Table 2:** Multivariable logistic regression of factors influencing outcome of laboratory-confirmed COVID-19 cases, Pavia, northern Italy, 21–28 February 2020 (n = 44)

**Outcome^a^**	**OR**	**95% CI**	**p value**
Lower probability of discharge from hospital			
Age > 65 years	0.043	0.004–0.504	0.012
Antiviral treatment	0.048	0.006–0.399	0.005
Severe disease^b^			
LDH level	1.090	1.022–1.163	0.008

CI: confidence interval; LDH: lactate dehydrogenase; OR: odd ratios.

^a^ Follow up as at 4 March 2020.

^b^ Patients were included in the mild disease group if they did not need high-flow oxygen support and in the severe disease group if they were provided with high-flow oxygen support.

In the supplementary materials we report detailed results of univariate and multivariable analysis. Particularly, Table S2 show results of model for discharge status, and Table S3 for severe disease. Table S4 reports the characteristics of patients who have received and who have not received antiviral treatment. Table S5 reports model for antiviral treatment. Eventually, Table S6 shows the characteristics of patients according to LDH levels.

Two patients received sub-intensive care. In these patients, time from symptom onset was 10 and 13 days, respectively and time from hospitalisation was 5 days. Three patients were admitted to the intensive care unit. Time from symptom onset in these patients was 10, 12, 25 days, respectively and time from hospitalisation was 4 days in two and 5 days in one patient. The deaths occurred 9 and 10 days, respectively, after the onset of symptoms and 3 days after hospitalisation. As at 4 March, 23 patients were still in the isolation room receiving standard care, including standard supportive care.

No bacterial infections were observed during the first week of observation. Two patients without known renal disease, developed kidney failure during hospital stay.

## Discussion and conclusions

Our early experiences from the COVID-19 pandemic showed significant morbidity in an aged population with high prevalence of coexisting illnesses. Older age and antiviral treatment were significantly associated with lower odds of hospital discharge; LDH levels > 300mg/dL were associated with higher odds of developing severe disease.

It is important to underline that the median age and prevalence of coexisting illnesses observed in our cohort were higher than those reported in studies from China [8,9], potentially leading to a more severe clinical course. Similar to other studies, the most common comorbidities were hypertension, chronic heart disease and diabetes mellitus [10].

A high number of our patients received antiviral treatment with LPV/r plus HCQ. However, patients who received antiviral treatment had lower odds of discharge. This association appears plausible because of the propensity of physicians to administer antiviral treatment to patients with more severe disease. The actual role of therapy, and the optimal treatment schedule for COVID-19 are still unclear. Doubts were raised regarding the clinical effectiveness of LPV/r [11] and several clinical trials are underway to evaluate the efficacy of other novel antivirals (e.g. remdesivir) and selective cytokine blockade (e.g. tocilizumab) [12].

Based on findings in the literature, most of our patients received antibiotic combination therapy, to cover both common and atypical pathogens [8]. Remarkably, no bacterial superinfections were observed during the first week of observation.

It is interesting to underline that among biological markers, heightened LDH levels were the only independent risk factors for severe disease. This finding was also reported in previous Chinese studies [8,13,14] as well as in an earlier study on Middle East respiratory syndrome (MERS) coronavirus [15]. It is well known that elevated LDH levels are suggestive of haematological malignancy and acute lung injury, such as Pneumocystis pneumonia (PCP) in HIV patients [16]. LDH levels might reflect tissue necrosis related to immune hyperactivity and thus relate to poor clinical outcome [17]. LDH levels may be a useful and easy to test parameter in order to identify patients at risk for severe respiratory failure.

The lymphocyte count is a parameter that has been described as linked to prognosis of COVID-19. Our laboratory data showed a low lymphocyte count in a considerable proportion of patients, a finding confirming previous reports [18-20]. Our results support the use of low absolute value of lymphocyte counts as a reference index in the diagnosis of COVID-19 [8,21].

Regarding the clinical characteristics of our cohort, fever was the most common symptom, this is similar to the results reported by a meta-analysis of studies performed in Chinese patients [22]. However, both cough and muscle weakness/fatigue were less frequent than in other studies. Although some studies demonstrated the presence of SARS-CoV-2 in stool samples [23] and that it might be associated with gastrointestinal symptoms [24], nausea and diarrhoea were rare in our patients. Further studies are needed to assess if these differences reflect peculiar clinical manifestation of the disease in the Italian context.

To date, more than 700 COVID-19 patients have been admitted to our hospital and further, more in-depth studies will follow. However, this picture of the first week of COVID-19 outbreak provides information regarding the disease outside China in a real life-setting of a country with very high case numbers. Although our study is limited by the small number of patients and by the short follow-up, our findings suggest that in a Caucasian aged population with high prevalence of coexisting illnesses, COVID-19 is characterised by notable morbidity.

## COVID19 IRCCS San Matteo Pavia Task Force

***ID Staff***

Raffaele Bruno, Mario U Mondelli, Enrico Brunetti, Angela Di Matteo, Elena Seminari, Laura Maiocchi, Valentina Zuccaro, Layla Pagnucco, Bianca Mariani, Serena Ludovisi, Raffaella Lissandrin Aldo Parisi, Paolo Sacchi, Savino FA Patruno, Giuseppe Michelone, Roberto Gulminetti, Domenico Zanaboni, Stefano Novati, Renato Maserati, Paolo Orsolini, Marco Vecchia.

Erika Asperges, Marta Colaneri, Alessandro Di Filippo

***ID Residents***

Margherita Sambo, Simona Biscarini, Matteo Lupi, Silvia Roda, Teresa Chiara Pieri, Ilaria Gallazzi, Michele Sachs, Pietro Valsecchi.

Emergency Care Unit

***ECU Staff*:** Stefano Perlini, Claudia Alfano, Marco Bonzano, Federica Briganti, Giuseppe Crescenzi, Anna Giulia Falchi, Roberta Guarnone, Barbara Guglielmana, Elena Maggi, Ilaria Martino, Pietro Pettenazza, Serena Pioli di Marco, Federica Quaglia, Anna Sabena, Francesco Salinaro, Francesco Speciale, Ilaria Zunino.

***ECU Residents*:** Marzia De Lorenzo, Gianmarco Secco, Lorenzo Dimitry, Giovanni Cappa, Igor Maisak, Benedetta Chiodi, Massimiliano Sciarrini, Bruno Barcella, Flavia Resta, Luca Moroni, Giulia Vezzoni, Lorenzo Scattaglia, Elisa Boscolo, Caterina Zattera, Tassi Michele Fidel, Capozza Vincenzo, Damiano Vignaroli, Marco Bazzini.

***Intensive Care Unit***

Giorgio Iotti, Francesco Mojoli, Mirko Belliato, Luciano Perotti, Silvia Mongodi, Guido Tavazzi

***Paediatric Unit***

Gianluigi Marseglia, Amelia Licari, Ilaria Brambilla

***Virology Staff***

Barbarini Daniela, Bruno Antonella, Cambieri Patrizia, Campanini Giulia, Comolli Giuditta, Corbella Marta, Daturi Rossana, Furione Milena, Mariani Bianca, Maserati Roberta, Monzillo Enza, Paolucci Stefania, Parea Maurizio, Percivalle Elena, Piralla Antonio, Rovida Francesca, Sarasini Antonella, Zavattoni Maurizio, Piero Marone.

***Virology Technical staff***

Adzasehoun Guy, Bellotti Laura, Cabano Ermanna, Casali Giuliana, Dossena Luca, Frisco Gabriella, Garbagnoli Gabriella, Girello Alessia, Landini Viviana, Lucchelli Claudia, Maliardi Valentina, Pezzaia Simona, Premoli Marta.

***Virology Residents***

Bonetti Alice, Caneva Giacomo, Cassaniti Irene, Corcione Alfonso, Di Martino Raffella, Di Napoli Annapia, Ferrari Alessandro, Ferrari Guglielmo, Fiorina Loretta, Giardina Federica, Mercato Alessandra, Novazzi Federica, Ratano Giacomo, Rossi Beatrice, Sciabica Irene Maria, Tallarita Monica, Vecchio Nepita Edoardo.

***Pharmacy Unit***

Monica Calvi, Michela Tizzoni

***Hospital Leadership***

Carlo Nicora, Antonio Triarico, Vincenzo Petronella, Carlo Marena, Alba Muzzi, Sara Cutti, Viola Novelli, Paolo Lago

***Data Unit***

Francesco Comandatore, Stefano Gaiarsa, Marco Rettani, Claudio Bandi, Alessandra Ferrari

## Supplementary Data

687000 Supplementary Tables
