# Supplementary material for: Clinical characteristics of coronavirus disease (COVID-19) early findings from a teaching hospital in Pavia, North Italy, 21 to 28 February 2020
Source: Euro Surveill. 2020 Apr 23;25(16):2000460. doi: 10.2807/1560-7917.ES.2020.25.16.2000460 (PMC7189652; doi:10.2807/1560-7917.ES.2020.25.16.2000460)
Supplement: Supplementary Tables [file 2200460_BRUNO_Supplementary_tables.pdf]

Disclaimer

This supplementary material is hosted by *Eurosurveillance* as supporting information alongside the article **Clinical characteristics of coronavirus disease (COVID-19) early findings from a teaching hospital in Pavia, North Italy, 21 to 28 February 2020** on behalf of the authors, who remain responsible for the accuracy and appropriateness of the content. The same standards for ethics, copyright, attributions and permissions as for the article apply. Supplements are not edited by *Eurosurveillance* and the journal is not responsible for the maintenance of any links or email addresses provided therein.

Table S1: Univariate analyses for disease severity and discharge status.

**Covariates**	**P-values for disease severity**	**P-values for discharge status**
Female sex	**0.084**	0.384
Age > 65	0.249	**0.004**
Respiratory frequency > 22	**0.092**	0.307
Fever	0.260	0.132
Cough	0.847	0.103
Dyspnea	0.560	0.788
Diarrhea	0.675	0.542
Fatigue	0.685	0.815
Interstitial pneumonia	0.722	**0.002**
Presence of comorbidities	0.279	**0.003**
Tumor	**0.049**	0.670
Heart disease	0.107	0.850
Hypertension	0.397	**0.033**
Diabetes	0.307	0.492
Lung disease	0.685	0.815
Hepatitis C virus	0.685	0.815
PF ratio < 260	**0.010**	0.423
Co2 < 35 mmHg	0.650	0.230
pH < 7.45	0.282	0.160
Leukopenia (WBC<5)	1	0.279
Lymphopenia	0.162	0.157
Thrombocytopenia	**0.025**	0.175
LDH levels	**0.012**	**0.033**
Creatinine > 1.5	0.293	0.852
CRP elevation	0.786	0.135
PCTI elevation	0.324	0.187
Antiviral therapy	**0.016**	**<0.001**
Antibiotic therapy	0.137	**0.0009**
Time from symptom onset	0.771	0.665
Time of hospitalization	0.394	0.243

Supplementary table S2. Multivariate model for discharge status

**Covariates**	Odds Ratio	95% Confidence Interval	p-value
Age > 65	0.043	0.004-0.504	**0.012**

Interstitial pneumonia	0.385	0.033-4.531	0.448
Presence of at least one comorbidity	0.141	0.005-4.018	0.252
LDH levels	0.998	0.984-1.013	0.875
Antiviral treatment	0.048	0.006-0.399	**0.005**
Antibiotic therapy	0.166	0.004-6.1445	0.330

Hypertension has not been included in the multivariate model to avoid collinearity with the presence of at least one comorbidity.

Supplementary table S3. Multivariate model for severe disease

**Covariates**	Odds Ratio	95% Confidence Interval	p-value
Female sex	0.058	0.001-1.994	0.1148
Respiratory frequency > 22	2.673	0.364-19.624	0.3337
Tumor	22.199	0.826-596.152	0.0648
Thrombocytopenia	1.050	0.099-11.128	0.9671
LDH levels	1.090	1.022-1.163	**0.0080**
Antiviral treatment	3.960	0.150-104.561	0.4098

PF ratio has not been included in the model because it was available in only 25 patients.

Supplementary table S4. Characteristics of patients who have received and who have not received antiviral treatment.

**Covariates**	**Antiviral treatment (n=31)**	**Not antiviral treatment (n=13)**	***p*-value**
Female sex	9 (29)	7 (53.8)	0.223
Age > 65	20 (64.5)	5 (38.4)	0.208
Respiratory frequency > 22	11 (35.5)	2 (15.4)	0.331
Fever	31 (100)	9 (69.2)	**0.008**
Cough	14 (45.2)	1 (7.7)	**0.041**
Dyspnea	8 (25.8)	2 (15.4)	0.720
Diarrhea	2 (6.5)	1 (7.7)	0.612
Fatigue	2 (6.5)	0 (0)	0.885
Interstitial pneumonia	26 (83.9)	5 (38.4)	**0.008**
Presence of comorbidities	21 (67.7)	7 (53.8)	0.596
Tumor	5 (16.1)	1 (7.7)	0.793
Heart disease	7 (22.6)	4 (30.8)	0.845
Hypertension	13 (41.9)	2 (15.4)	0.178
Diabetes	4 (19.9)	3 (23.1)	0.697
Lung disease	1 (3.2)	1 (7.7)	0.885
Hepatitis C virus	1 (3.2)	1 (7.7)	0.885
PF ratio < 260	10 (32.2)	0 (0)	0.379
Co2 < 35 mmHg	14 (45.2)	2 (15.4)	0.590
pH < 7.45	4 (19.9)	2 (15.4)	0.261
Leukopenia (WBC<5)	17 (54.8)	5 (38.4)	0.509
Lymphopenia	30 (96.8)	9 (69.2)	**0.035**
Thrombocytopenia	16 (51.6)	2 (15.4)	**0.058**

LDH >300	13 (41.9)	1 (7.7)	**0.093**
Creatinine > 1.5	2 (6.5)	0 (0)	0.925
CRP elevation	7 (22.6)	1 (7.7)	0.522
PCTI elevation	6 (19.4)	2 (15.4)	0.959
Antibiotic therapy	29 (74.2)	3 (23.1)	**<0.0001**
Mean time from symptom onset	15.2 ± 8	14 ± 5	0.549
Mean Time of hospitalization	8.4 ± 2.0	7.2 ± 2.5	0.133

Supplementary table S5. Multivariate analysis for antiviral treatment

**Covariates**	Odds Ratio	95% Confidence Interval	p-value
Fever	0.00	-	0.995
Cough	5.302	0.318- 88.250	0.244
Interstitial pneumonia	1.476	0.087-25.036	0.787
Lymphopenia	0.00	-	0.996
Thrombocytopenia	4.278	0.245-74.697	0.319
LDH>300	0.00	-	0.995
Antibiotic	5.059	0.120-213.554	0.395

Supplementary table S6. Characteristics of patients according to LDH levels.

**Covariates**	**LDH > 300 (n=15)**	**LDH< 300 (n=29)**	***p*-value**
Female sex	4 (26.7)	10 (34.5)	0.845
Age > 65	10 (66.7)	14 (48.3)	0.383
Respiratory frequency > 22	4 (26.7)	8 (27.6)	0.771
Fever	13 (86.7)	24 (82.8)	0.882
Cough	7 (46.7)	8 (27.6)	0.346
Dyspnea	5 (33.3)	5 (17.2)	0.405
Diarrhea	1 (6.7)	1 (3.4)	0.780
Fatigue	0 (0)	2 (6.9)	0.780
Interstitial pneumonia	13 (86.7)	17 (58.6)	**0.093**
Presence of comorbidities	10 (66.7)	17 (58.6)	0.845
Tumor	2 (13.3)	4 (13.8)	0.674
Heart disease	4 (26.7)	7 (24.1)	0.849
Hypertension	7 (46.7)	8 (27.6)	0.346
Diabetes	2 (13.3)	5 (17.2)	0.923
Lung disease	0 (0)	2 (6.9)	0.780
Hepatitis C virus	0 (0)	2 (6.9)	0.780
PF ratio < 260	4 (26.7)	11 (37.9)	0.327
Co2 < 35 mmHg	4 (26.7)	11 (37.9)	0.327
pH < 7.45	0 (0)	6 (20.7)	**0.088**
Leukopenia (WBC<5)	8 (53.3)	12 (41.4	0.656
Lymphopenia	13 (86.7)	24 (82.8)	0.882
Thrombocytopenia	8 (53.3)	10 (34.5)	0.369
Creatinine > 1.5	1 (6.7)	1 (3.4)	0.780
CRP elevation	3 (20)	5 (17.2)	0.804
PCTI elevation	3 (20)	5 (17.2)	0.873
Antiviral therapy	13 (86.7)	17 (58.6)	**0.093**

Antibiotic therapy	12 (80)	19 (65.5)	0.483
Mean time from symptom onset	16.7 ± 7.4	13.4 ± 4.9	**0.083**
Mean Time of hospitalization	8.1 ± 2.1	6.9 ± 2.5	0.110
